# Percutaneous ozone nucleolysis for lumbar disc herniation

**DOI:** 10.1007/s00234-018-2083-4

**Published:** 2018-09-11

**Authors:** Mohamed Ezeldin, Marco Leonardi, Ciro Princiotta, Massimo Dall’olio, Mohammed Tharwat, Mohammed Zaki, Mohamed E. Abdel-Wanis, Luigi Cirillo

**Affiliations:** 1 Department of Diagnostic and Interventional Radiology, Faculty of Medicine, Sohag University, Sohag, Egypt; 2 Neuroradiology Unit, Bellaria Hospital & IRCCS Institute of Neurological Sciences, Bologna, Italy; 3 DIMES, Department of Specialty, Diagnostic and Experimental Medicine, University of Bologna, Bologna, Italy; 4 Department of Orthopaedic Surgery, Faculty of Medicine, Sohag University, Sohag, Egypt

**Keywords:** Ozone, Lumbar disc herniation, Pain, Oswestry Disability Index

## Abstract

**Purpose:**

All percutaneous minimally invasive disc treatments are typically indicated to contained disc herniations. Our study’s aim is to evaluate prospectively the efficacy of ozone nucleolysis in the treatment of either contained or uncontained lumbar disc herniations.

**Methods:**

Fifty-two patients, aged 27–87 years, with symptomatic herniated lumbar discs, without migration, sequestration, or severe degenerative disc changes, who failed conservative treatment, were included in our study. The patients underwent fluoroscopic-guided intradiscal oxygen-ozone mixture injection (5 ml) at a concentration of 27–30 μg/ml and periradicular injection of the same O_2_-O_3_ mixture (10 ml), steroid (1 ml), and local anesthetic (1 ml). Clinical outcomes were evaluated, based on the Oswestry Disability Index (ODI) and pain intensity (0–5) scale results, obtained initially and at 2- and 6-month controls. Our results were analyzed by ANOVA and chi-squared (*χ*^2^) tests.

**Results:**

Our initial results obtained at 2-month control were promising, indicating a significant decrease in pain disability and intensity in 74% (37) and 76% (38) of the patients respectively, and minimally increased to 76% (38) and 78% (39) at 6-month control (*P* < 0.001 and CI 99.9%**)**. The mean preprocedure ODI and pain intensity scores were 35 ± 14.36 and 2.38 ± 0.90, respectively, which were reduced to 19.36 ± 13.12 and 1.04 ± 0.92 at 6-month control. Our failure had been mostly related to long symptoms duration of more than 1 year. No complications were recorded.

**Conclusion:**

Ozone nucleolysis is a safe cost-effective minimally invasive technique for treatment of contained and uncontained lumbar disc herniations.

## Introduction

Lumbar disc herniation is one of the most common causes of low back pain and/or radiculopathy. The pathogenesis of lumbo-radicular pain due to lumbar disc herniation is likely related to mechanical and or inflammatory factors [1, 2]. The natural history of symptomatic disc herniation is favorable, with the majority (80–90%) of patients showing improvement within 6–12 weeks. However, relief of pain and disability during this period is essential [3]. The first-line treatment for pain management due to disc herniation is conservative. If conservative treatment, including rest, medications, and physical therapy, failed to relieve the pain, disc decompression is considered. The results of surgical treatment are suboptimal, and surgery harbors significant morbidity and potential complications [4].

Therefore, many alternative minimally invasive disc decompression techniques have been developed over the last few decades to reduce the need for surgery. Nowadays, surgery is confined for treatment of patients with a progressive neurological deficit, cauda equina syndrome, and severe intolerable pain. Minimally invasive techniques include percutaneous mechanical, laser or radiofrequency coblation (nucleoplasty) disc decompression, and chemonucleolysis using gelified alcohol “Discogel®” or oxygen-ozone mixture [4–6]. The oxygen-ozone chemonucleolysis is as effective as other percutaneous disc decompression techniques, which has a high therapeutic success rate (70–80%) with the lowest cost and complications [7–9].

Several experimental studies investigated the mechanisms of action of intradiscal and periganglionic injection of an oxygen-ozone mixture. Ozone has a powerful oxidizing effect on the proteoglycans of the nucleus pulpous, resulting in matrix dehydration, degeneration, and subsequent reduction of the herniating disc volume. Small-volume reduction can result in a significant drop in intradiscal pressure, thereby alleviating compression on the nerve roots and surrounding vessels with consequent reduction of venous stasis, nerve roots edema, and hypoxia. Also, it increases tissue oxygenation as it increases the concentration of 2,3-diphosphoglycerate within the red blood cells. Moreover, ozone has also a potent analgesic and anti-inflammatory actions through inhibition of synthesis and release of pro-inflammatory cytokines, prostaglandins E2, bradykinins, and stimulation of release of anti-inflammatory cytokines [7, 8, 10–17].

All percutaneous minimally invasive disc treatments are typically indicated for the treatment of contained disc herniations [4, 6]. The aim of our prospective study is to evaluate the efficacy of oxygen-ozone nucleolysis in the management of pain and disability of either contained or uncontained lumbar disc herniations.

## Materials and methods

### Study design and participants

This is a prospective clinical study of 6-month follow-up period, conducted on patients treated with oxygen-ozone chemonucleolysis, at Neuroradiology unit, Bellaria Hospital, IRCCS Institute of Neurological Sciences, Bologna. Italy, between December 2016 and April 2017. The study was performed in accordance with our institutional and/or national ethical and health guidelines with written informed consents were obtained from each participant prior to the treatment. All patients underwent a neurological evaluation with reviewing their magnetic resonance imaging (MRI) to confirm nerve roots or thecal compression. Out of all patients treated by O_2_-O_3_ chemonucleolysis, 52 patients who met our inclusion criteria were enrolled in this study with two of them lost to follow-up. The mean age of the remaining 50 patients was 54.5, ranging from 27 to 87 years at the time of the procedure. Demographic, clinical, and imaging data of the patients are shown in Table 1.

**Table 1:** Demographic data of the patients

Patient characteristics	Data
Total population	*N* = 50
Mean age (years) ± SD	54.5 ± 12.9
Age distribution	
Young adult/middle age/old age	5/27/18
Gender	
Male/female	25/25
Mean pain duration (months) ± SD	19.1 ± 17.3
History duration	
Short; less than 1-year/long	29/21
Main complain	
Sciatica/lumbalgia	44/6
Disability symptoms	
Mild/moderate/severe/extreme/bedridden	9/26/13/2/0
Lesions morphology	
Bulges(s)/protrusion(s)/extrusion(s)/mixed	11/23/11/5
Involved disc levels	
One/two/three	30/17/3
Disc degeneration	
Mild; “Pfirrman grading III”	12
Moderate; “Pfirrman grading IV”	38
Modic changes	
Type 0/I/II/III	29/19/2/0

Inclusion criteria included patients with bulging, protruded, or extruded herniated lumbar discs, low back pain and/or radiculopathy, neuroradiological findings correlating with the clinical symptoms, and failure of conservative medical and/or physical therapies of at least 2-month duration. Exclusion criteria included patients with major or progressive neurological deficit, cauda equine syndrome, large migrated or sequestrated disc herniations, severe disc degeneration with height reduction of more than two thirds; Pfirrman grading of V, asymptomatic disc herniations, structural spine abnormalities such as spinal stenosis or spondylolisthesis, failed back surgery, spinal tumors or fractures at the same level to be treated, active infections, active hyperthyroidism, heart failure, hemorrhagic diathesis, and pregnant patients.

### Oxygen-ozone nucleolysis technique

#### Patient preparation

The procedure, the benefits, and associated potential complications were explained to all patients. All patients underwent routine blood analysis and electrocardiogram before the procedure, to rule out the presence of coagulation defects, signs of infection or heart disease, and to recognize the presence of diabetes (in which the procedure is performed without injecting steroids). Patients did not receive any prophylactic antibiotics, and the procedure was done without general or local anesthesia to avoid any masked accidental nerve roots contact.

#### Injection technique

All procedures were performed by three senior interventional neuroradiologists, under fluoroscopic guidance using a digital angiography equipment (Philips Allura Xper FD), with the patient in lateral decubitus position, resting on the non-painful side. The lumbar region was thoroughly disinfected, and sterile drapes were applied. At first, the lateral projection was obtained and alignment of the end plates of the concerned disc space was performed, through cranial and caudal angulations of the C-arm to clearly open the disc space. Then, the C-arm was rotated at an angle of 40°–45°, so that the facet joint superimposed on the posterior third of the disc space, can produce the so-called Scotty dog appearance. On the side of main pain location, needle puncture was performed through paravertebral oblique approach using a 22-gauge, 15-cm Chiba needle, modified with the addition of three radial holes near the tip to facilitate the distribution of the gas inside the nucleus pulposus, a 20-cm needle is used for obese patients.

**Fig. 1:**
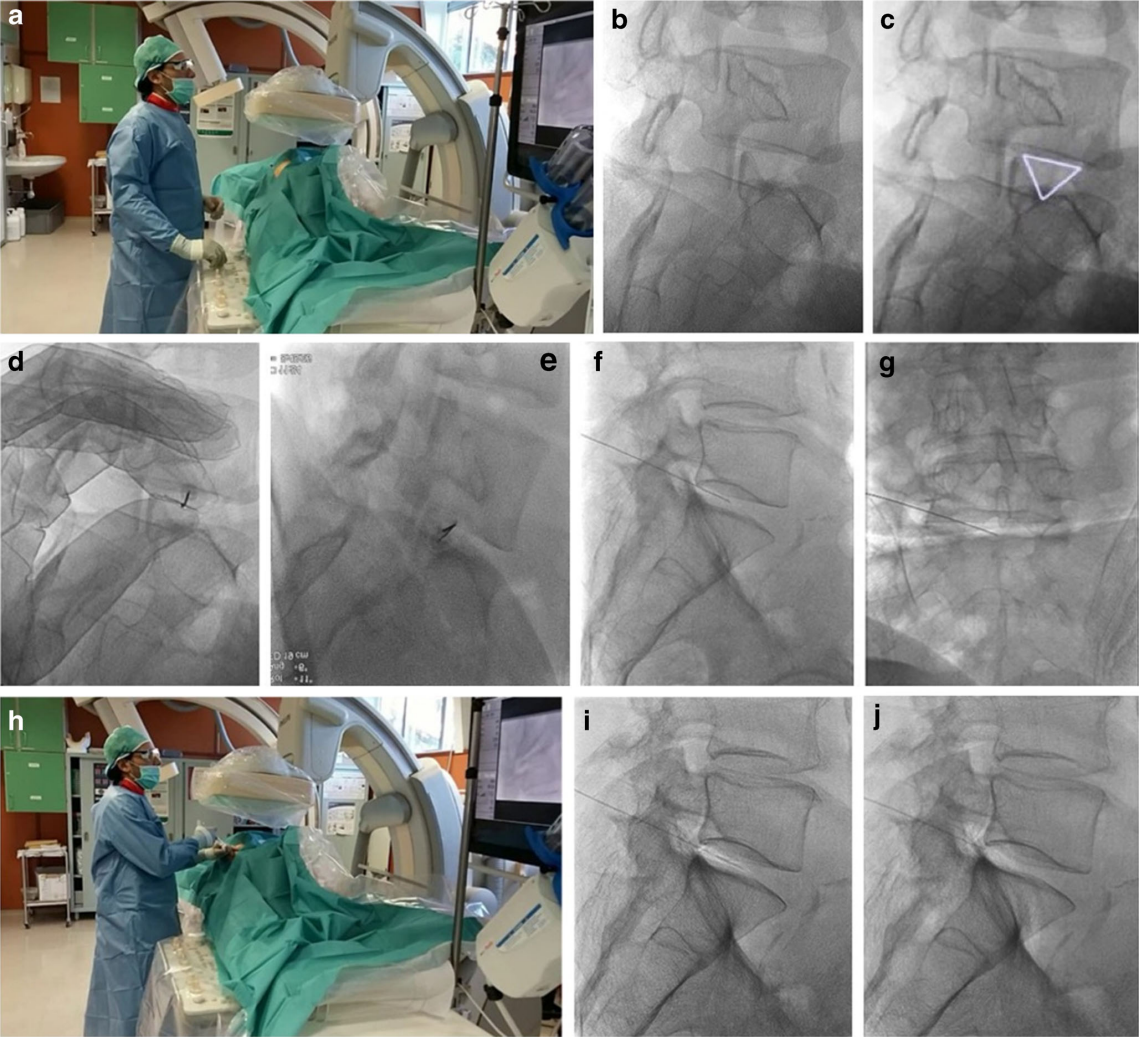
Demonstration of the injection technique. **a** Photographic image shows the operator (**a**) starting to adjust C-arm positioning, required for needle guidance and insertion. **b**, **c** Oblique fluoroscopic views at the level of L5-S1 space, showing the so-called Scotty dog appearance with superimposed virtual (white) triangle, through which the needle should be inserted. **d**, **e** Oblique fluoroscopic images, obtained during and after needle insertion and advancement, showing the needle appearing as a small radiolucent line projected over the concerned disc space. **f**, **g** Lateral and anteroposterior fluoroscopic views, obtained prior to oxygen-ozone mixture injection, showing the needle tip inside the center of the concerned disc space. **h** Another photograph for the operator demonstrates the injection procedure of an oxygen-ozone mixture. **i**, **j** Lateral fluoroscopic views obtained after oxygen-ozone mixture injection, demonstrating the injected intradiscal (**i**) and intraforaminal (**j**) oxygen-ozone gas as a faint opacity around the needle tip

In the oblique projection, the needle was inserted just anterior and lateral to the superior articular process of the inferior vertebra (i.e., “Scotty dog” ear) at the direction of the X-ray beam. Once the needle is inserted through the paravertebral muscles for a short distance, C-arm was resumed to the lateral position and needle advancement continues until reaching a final position at the center of the concerned disc. Then, the anteroposterior projection was used to check the proper needle position within the nucleus pulposus and can be corrected if needed. In case of L5-S1 disc space, more cranio-caudal angulation and oblique tilting of the C-arm was further required due to an encroachment of the iliac wing on the disc space. The aim was to send the facet joint more posteriorly to create a virtual inverted narrow triangle bordered by the iliac wing, the superior articular process of S1, and the lower L5 surface, through which the needle was introduced **(**Fig. 1**)**.

Under real-time fluoroscopic monitoring at lateral view projection, 5 ml of an oxygen-ozone mixture at a concentration of 27–30 μg/ml was obtained using a medical ozone generator (Ozo2 Futura, Alnitec, Italy) and injected into the disc. In case of extruded disc herniation, more dose is required (up to 10 ml), as part of the injected mixture is leaked into the epidural space. Then, the needle was withdrawn into the intervertebral foramen and 10 ml of the O_2_-O_3_ mixture was injected around the nerve root, followed by periganglionic injection of 1 ml of steroid (triamcinolone acetonide, 40 mg) associated with 1 ml of local anesthetic (bupivacaine, 5 mg).

#### Post-procedure care

The patients remained in lateral decubitus for 30 min and then in supine decubitus for at least 2 h. Patients were instructed for relative bed rest for the first day after the procedure and home rest for at least the next 2 days. The patients were asked to limit physical work and avoid any lifting, prolonged seating, bending, or twisting the spine during the following 2 weeks after the procedure. Physical therapy was advised 1 month later with the emphasis on lumbar exercises. Heavy lifting is avoided on the next 6 months.

### Outcome evaluation

Our primary outcome was an assessment of pain disability using the Oswestry Disability Index (ODI) questionnaire [18]. ODI is a percentage score (ranges from 0% = no disability to 100% = maximum disability, with a lower score that indicates less severe disability). The score is calculated from a 10-item questionnaire, one item for pain and the other items to assess the pain impact on daily life activities such as personal care, lifting, walking, sitting, standing, sleeping, sex life (if applicable), social life, and traveling. Pain intensity as a secondary outcome was estimated using a simple descriptive (0–5) pain scale included in the ODI questionnaire. This score ranges from 0 = “no pain” to 5 = “worst possible pain.” The ODI and pain intensity values were recorded initially and at postprocedure intervals of 2 and 6 months. A clinically significant outcome was defined as a reduction in the preoperative ODI values or pain intensity scores of at least 30% during follow-up, in accordance with literature recommendations [19]. The outcome was graded according to the percentage of reduction into poor (0–29%), average or fair (30–49), good (50–74), and excellent outcome if the reduction was equal or more than 75%. Patients were considered to have a failure if graded as poor.

### Statistical analysis

Data were collected and analyzed using SPSS Statistics Program, version 20. Quantitative data were expressed as mean ± standard deviation, percentages, or numbers. Repeated measures ANOVA test was used to compare the pre- and posttreatment ODI data. If the test showed a statistical difference, a paired *t* test with Bonferroni’s correction was used to perform pairwise comparisons. Analyses of the impact of patients’ characteristics on the response were done using the chi-squared (*χ*^2^) test or Fisher’s exact test, when necessary. *P* < 0.05 was considered statistically significant in all analyses.

## Results

### Patients’ demographics

Our study included 52 patients. Two patients were lost to follow-up, and results were obtained in 50 (25 males, 25females) patients. All patients were operated on at one level (30 patients) or two levels (17 patients), except for three cases which were operated on at three levels. The included levels extended from L3 down to S1 with main distribution at L4-L5 and/or L5-S1 disc spaces (84%; 42 patients).

### Outcome analyses

Our results were initially obtained at 2 months follow-up, and not before to avoid the effect of the periradicular corticosteroid, used after chemonucleolysis. The mean preprocedure ODI score was 35 ± 14.36, which is reduced to 20.16 ± 11.68 at the first control and to 19.36 ± 13.12 at the last control, as shown in Table 2. The mean reduction and percentages of improvement for ODI at the initial 2-month control was 14.84 and 42.40%, respectively, and was 15.64 and 44.69%, respectively, at 6-month control.

**Table 2:** ODI scores during the follow-up period

ODI score	Minimum	Maximum	Mean	Std. deviation
Preprocedure	10	80	35.00	14.36
2-month control	0	54	20.16	11.68
6-month control	0	60	19.36	13.12

The average pain intensity score was 2.38 ± 0.90 (minimum 1–maximum 5) prior to the procedure, 1.32 ± 0.81 at 2 months (minimum 0–maximum 3) and 1.04 ± 0.92 (minimum 0–maximum 3) at 6 months. There were statistically significant differences in different ODI and pain scores obtained before and after the treatment (*P* < 0.001 and CI 99.9%).

**Fig. 2:**
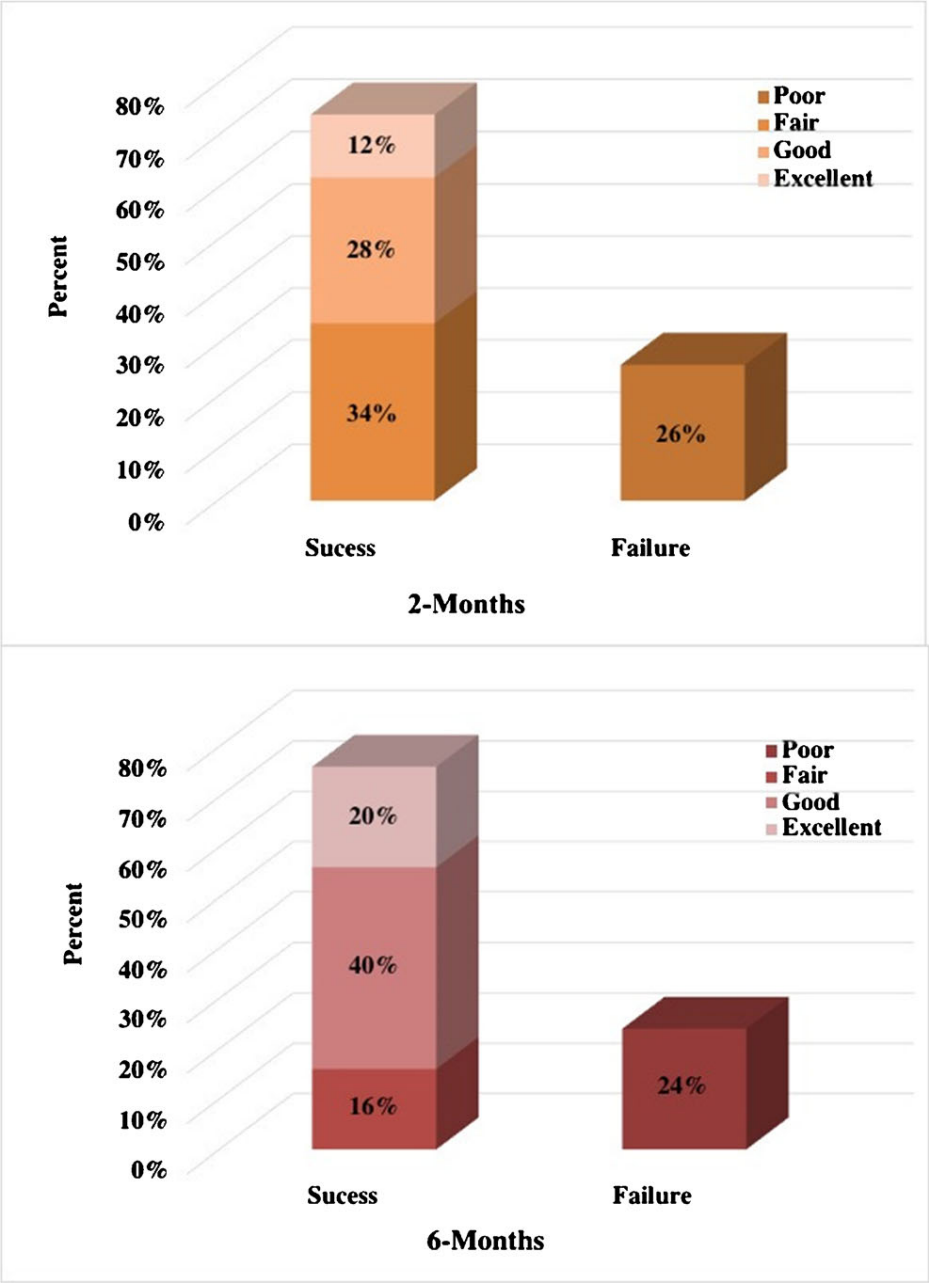
Two graphs show outcomes of pain disability during the follow-up

**Fig. 3:**
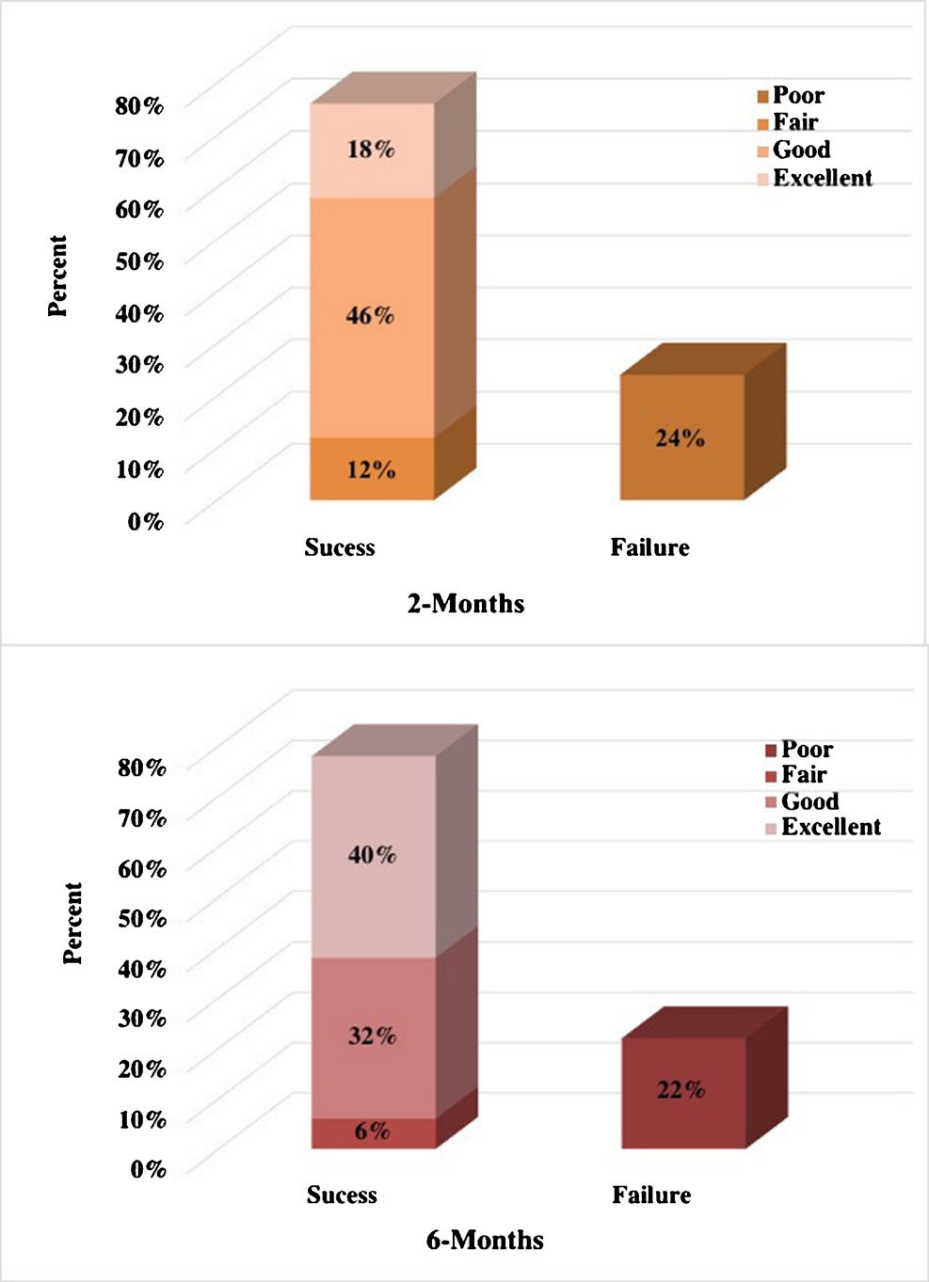
Two graphs show outcomes of pain intensity during the follow-up

The percentage of patients reporting significant improvement in disability symptoms, according to ODI scores was 74% (37 patients) at the time of 2-month follow-up and minimally increased to 76% (38 patients) at 6-month follow-up. Excellent and good outcomes were noted in 60% (30 patients), fair in 16% (8), and poor in 24% (12) of the patients at the last control (Fig. 2). According to pain intensity score, significant decreases in pain intensity was found at 76% (38 patients) at the initial control and minimally increased to 78% (39 patients) at 6-month control, which was good to excellent in 72% (36) and fair at 6% (3) of the patients (Fig. 3). Occasional administration of a simple analgesia was encountered in few patients with a positive response, especially those of a fair outcome.

The statistical analyses of the impact of patients’ characteristics on the outcome were studied. The percentage of success was higher with a statistically significant difference in patients with a short history of symptoms (less than 1 year), which was 86.2% compared to 61.9% in patients with a long history (*P* < 0.05). On the other hand, there was no significant difference between the responders and non-responders in terms of the age, gender, the degree of pretreatment disability, the type of the herniated disk, the injection level, the number of operated discs, and mildly or moderately degenerated discs, as shown in Table 3. Patients with poor outcome were referred for neurosurgical consultation, and two patients of them were planned for surgical decompression.

**Table 3:** Primary outcome analyses according to patient characteristics

Patient characteristics		Success	Failure	*p* value (*χ*^2^) and fissure test
Total population		*n* = 38 (76%)	*n* = 12 (24%)	
Age (years)		M = 54.34	M = 55.33	
Age groups	Young adult/middle age/old age	4/18/13	1/7/6	0.891^a^–0.888
Gender	Male/female	18/18	6/8	0.508
History	Short; < 1 year	24	4	
	Long; > 1 year	12	10	0.047^*^
Pain duration (months)		M = 15.94	M = 28.64	
Disc lesions morphology	Bulges(s)	7	4	0.501^a^–0.575
	Protrusion(s)	16	7	
	Extrusion(s)	10	1	
	Mixed	3	2	
Operated side	Right/left	18/18	9/5	0.128
Disc levels	One/two/three	23/10/3	7/7/0	0.145^a^–0.177
Disc degeneration	Mild/moderate	7/29	0/14	0.926^a^–1.000
Modic changes	0/I/II	21/2/13	7/0/7	0.979
Preprocedure ODI		M = 36.47	M = 30.33	

*n* number, *M* mean

*The chi-square statistic is significant at *P* value < 0.05

^a^Chi-square results were insufficient, and exact fissure test was done

### Complications

No minor or major complications as nerve roots injuries, bleeding, or discitis were encountered during, immediately after the procedure, or during follow-up.

## Discussion

Oxygen-ozone chemonucleolysis is one of the minimally invasive disc treatments that is commonly used in Europe and Asia for the treatment of disc herniation since the late 1990s [8]. Ozone (O_3_) is a colorless unstable tri-atomic form of oxygen with an irritant pungent odor which is normally present in the atmosphere as a gas layer that protects the living organisms from the harms of ultraviolet rays. Medical ozone is administered in the form of an O_2_-O_3_ mixture, at non-toxic concentrations, not exceeding 40 μg of O_3_ per ml of oxygen and prepared through conversion of pure oxygen (O_2_) into ozone (O_3_) using special medical generators, that can adjust ozone concentration as required [8, 10, 20, 21].

In our series, the procedure was performed under fluoroscopic guidance as a simple, rapid, and real-time control of the puncturing needle and gas distribution was allowed. The procedure can also be conducted under CT guidance with possible less X-ray exposure for the operator. The patients received single-session treatment of intradiscal ozone injection with concomitant periradicular infiltration of the oxygen-ozone mixture, steroid, and local anesthetic, as combined injection of these materials, had more cumulative effects with a more enhanced outcome than the use of ozone or steroid alone [22–25].

In a previous randomized controlled trial (RCT), Andreula et al. concluded that combined treatment of intradiscal ozone and periradicular injection of oxygen-ozone, steroid, and local anesthesia has a significant cumulative effect at 6-month follow-up, with a higher statistically significant success rate in 78.3% of the patients compared to a success rate of 70.3% in those treated with intradiscal injection of ozone alone [10]. Also, Gallucci et al. reported a success rate of 74% in a group of patients treated with combined intradiscal and interaforaminal injections of oxygen-ozone, steroid, and local anesthesia versus a success rate of 47% in steroid and local anesthesia group, concluding that combined ozone and steroid treatment was more effective at 6-month control [23].

Transforaminal epidural steroid injection (TFESI) is a well-known adjunct in all percutaneous disc treatments, and triamcinolone is one of the most effective steroids utilized for this purpose. There are, however, potential neurological risks of spinal cord or brainstem infarcts when particulate steroids are used. These risks are greater with cervico-thoracic or lumbar injections above L3 with only 1% of people having a risk below this level. Non-particulate steroids such as dexamethasone are recommended for usage at higher levels [26]. In our series, all treated levels were distributed from L3–4 down to L5-S1 disc space.

The injected O_2_-O_3_ mixture concentration was 27–30 μg ozone/ml of oxygen because such concentration was the best one that proved, by experimental studies, to dry out the nucleus pulposus proteoglycans and reduce the inflammation at disc/nerve root conflict [13, 20, 22]. The maximum ozone concentration applied safely for intradiscal and periganglionic injections is 40 μg/ml of oxygen. A higher concentration can induce an acute non-controlled oxidative stress that can exceed the capacity of anti-oxidant enzymes with subsequent accumulations of hydrogen peroxides, which can cause cell membrane degradation [8, 10].

On the basis of our results, the 6-months outcome was satisfactory and showed significant improvement of disability symptoms in 76% of the patients, mean reduction of ODI score of 15.64 points, and pain relief in 78% of the patients. There were no relevant complications during follow-up period. Our failure has been mostly related to long symptoms duration of more than 1 year. We did not find any other significant prognostic factors to be correlated to our success or failure. Our clinical results were similar to those obtained from other similar studies [7, 10, 23–25, 27, 28], with a little difference in the included inclusion criteria or outcome measures. The meta-analysis of these studies reported ozone effectiveness in the range of 70 to 80%, a mean reduction of ODI score of 14.1points, and very rare incidence of complications, less than 0.1% [9].

Our results were also comparable to those obtained from other percutaneous disc decompression techniques as well as surgical treatments [4, 6, 9, 29]. However, ozone chemonucleolysis has the advantage of being less invasive, as it uses smaller (22 gauge) needles with very low complications rate (< 0.1%). In addition, it is a single-shot, 1-day hospitalization treatment with a lower cost compared to other alternative minimally invasive disc treatments, such as Discogel® chemonucleolysis, radiofrequency nucleoplasty, Dekompressor® discectomy, or multiple intensified physiotherapy sessions. Moreover, the treatment is effective with broad inclusion criteria that include all types of disc lesions with no sequestrated fragments and even at multiple levels. Also, the treatment can be administered more than one time, without preclusion of further surgical options [7–9, 25].

Percutaneous decompression techniques are typically indicated for small to medium contained disc herniations. Containment is essential in some percutaneous treatments especially the mechanical and thermal (laser and radiofrequency) decompression techniques, as they are based on the principle that the contained disc is a closed hydraulic space. Therefore, removal of a part of its nucleus pulposus will allow subsequent reduction of intradiscal pressure and elastic recoil of the herniated portion [4, 6, 29].

**Fig. 4:**
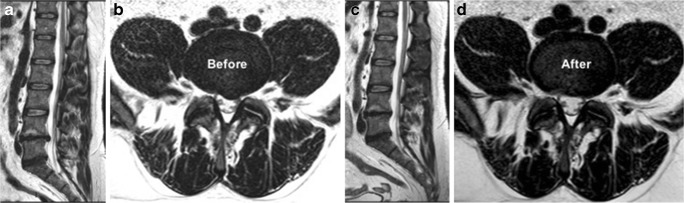
MR images of a 71-year- old man complaining of left lumbosciatica of 2-year duration and showed a significant clinical and radiological improvement after intradiscal O_2_-O_3_ injection. **a**, **b** Preprocedure **s**agittal and axial MRI T2-weighted images showing an extruded left para-centeral disc herniation at the level of L4–5, compressing the dural sac and the descending nerve roots. **c**, **d** Follow-up sagittal and axial MRI images obtained 6 months after the procedure showing near-complete resolution of the extruded disc hernia

**Fig. 5:**
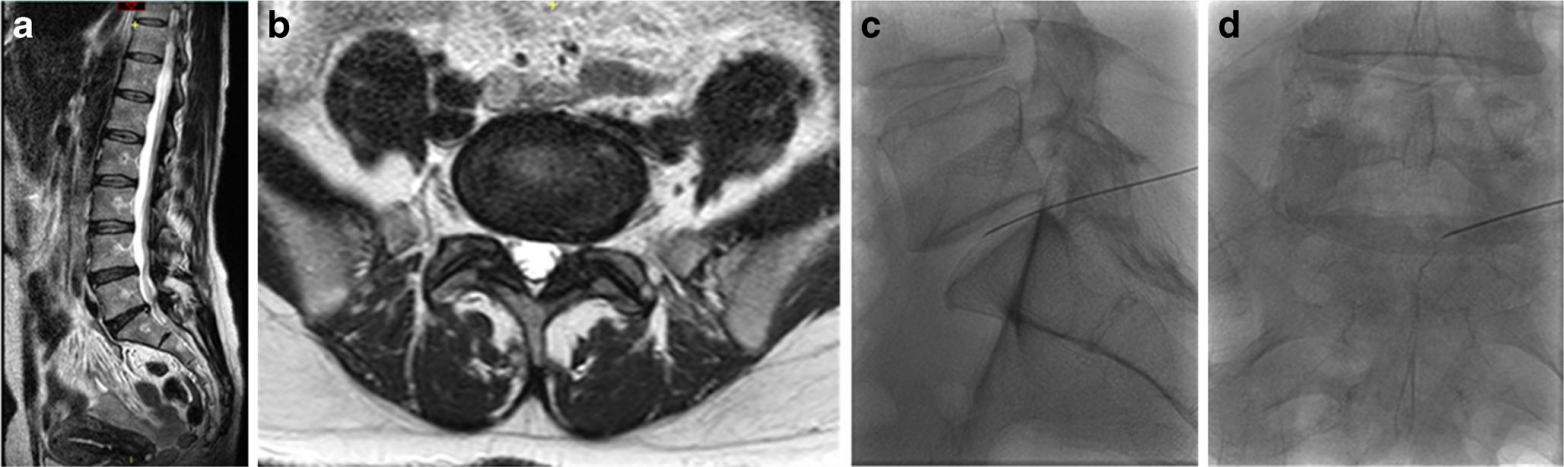
A 52-year-old women complaining of left lumbosciatica of 1-year duration and treated by intradiscal injection of an O_2_-O_3_ mixture. **a** MRI T2-weighted sagittal image, showing diffuse degenerative changes, with an associated bulging disc at L5-S1 level. **b** Axial MR T2-weighted image of the same patient shows diffuse disc bulge at the aforementioned level, more inclined to the left side with encroachment on the ipsilateral neural foramen. **c**, **d** Lateral and anteroposterior fluoroscopic images displaying the needle tip inside the disc center

**Fig. 6:**
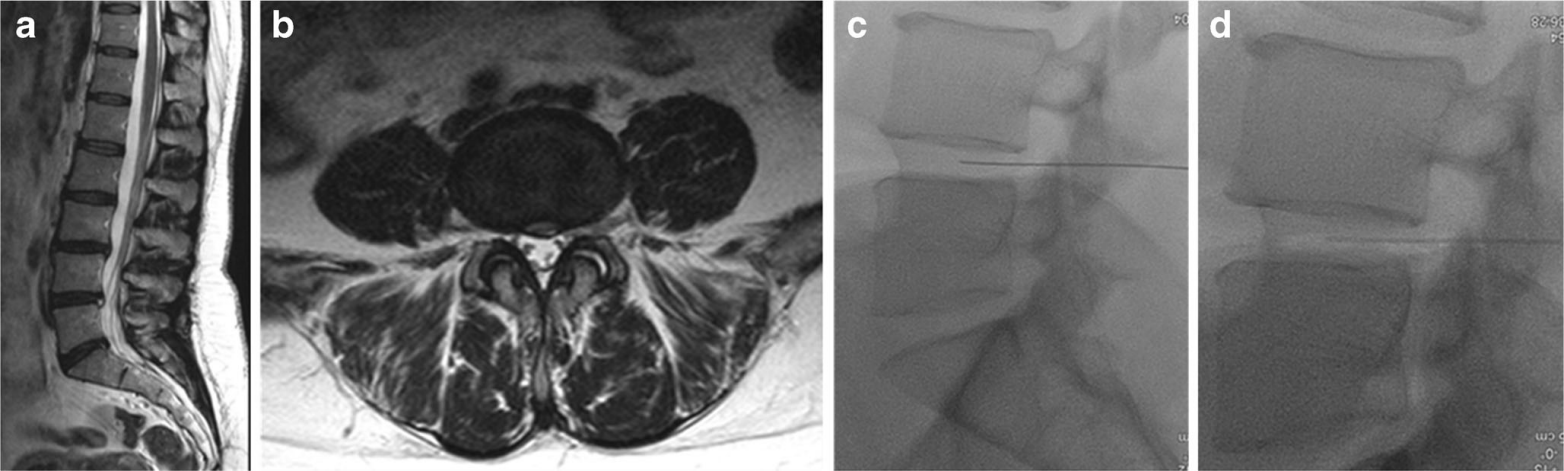
A 43-year-old women presented with lumbalgia and treated by of O_2_-O_3_ discolysis. **a** MRI T2-weighted sagittal image shows diffuse degenerative changes with small disc protrusion at the level of L4–5, associated with a high signal annular tear. **b** The corresponding axial MRI T2-weighted image shows a left para-median disc protrusion, minimally indenting the dural sac, at the aforementioned level. **c**, **d** Lateral fluoroscopic images of the same patient show the needle placed inside the disc center as well as the distribution of O_2_-O_3_ mixture (**d**) after the injection that displayed as a faint white opacity inside the disc and epidural space

**Fig. 7:**
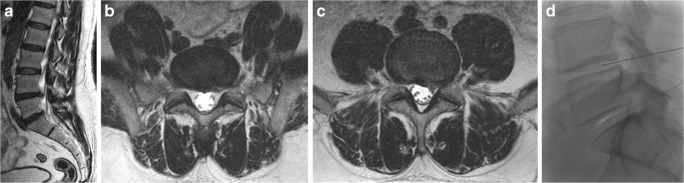
A 57-year-old man complaining of left lumbosciatica of 10-month duration and treated by O_2_-O_3_ chemonucleolysis. **a** Sagittal MRI T2-weighted image shows diffuse degenerative disc changes with small disc protrusions at the lower two lumbar levels. **b**, **c** The corresponding axial MRI T2-weighted images show left postero-lateral protrusions; at the levels of L4–5 and L5-S1, more evident at the latter one. **d** Lateral fluoroscopic image of the same patient shows the needles inside the center of corresponding discs, with ozone gas displayed as white linear opacity at L5–1 disc space

**Fig. 8:**
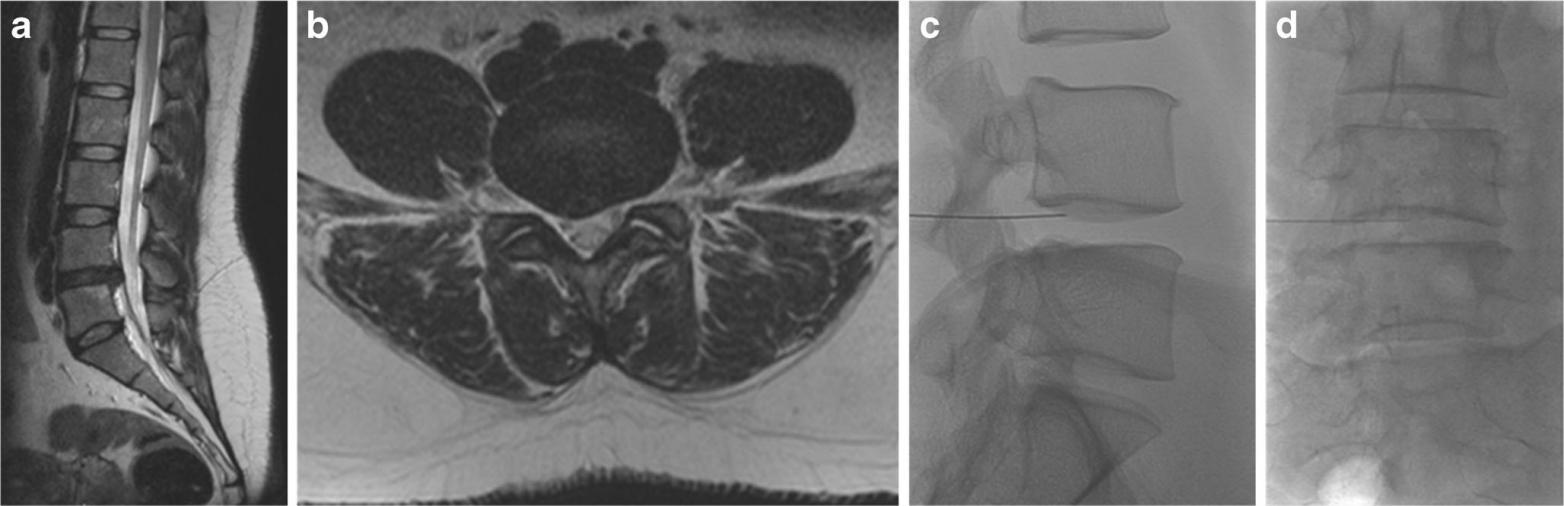
A 34-year-old women complaining of right lumbosciatica of 2-year duration and treated with ozone chemonucleolysis. **a** MRI T2-weighted sagittal image showing an extruded disc herniation at the level of L4–5, with pressure on the dural sac. **b** The corresponding axial MR T2-weighted image shows the disc herniation located at a right para-median site, compressing the dural sac as well as the traversing L5 nerve roots. **c**, **d** Lateral and anteroposterior fluoroscopic images showing the needle inside the disc center

**Fig. 9:**
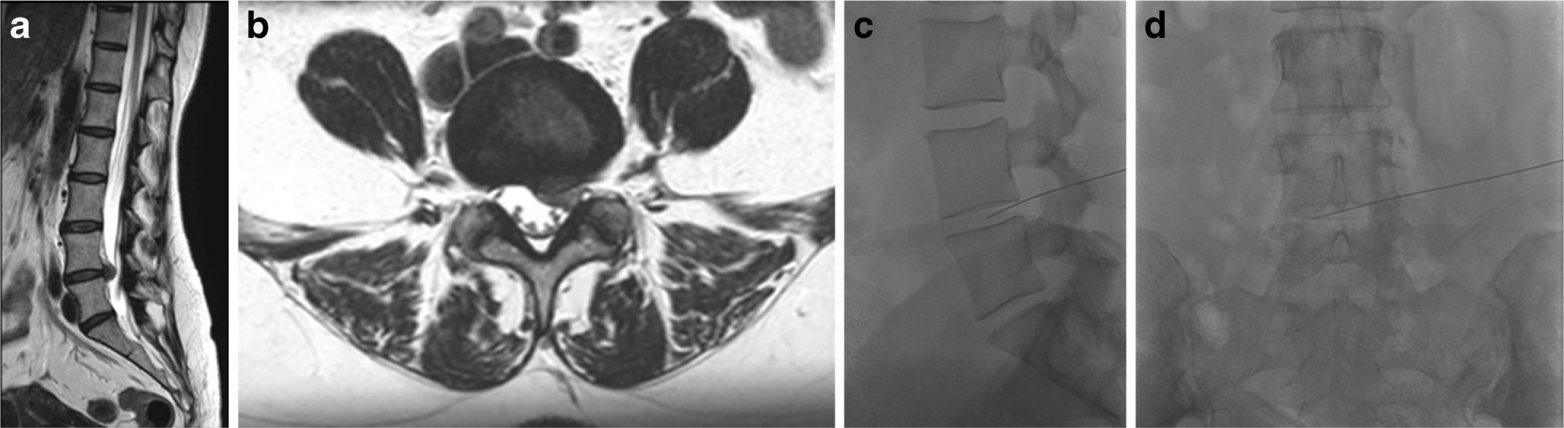
A 59-year-old women complaining of left lumbosciatica of 6-month duration and treated with ozone chemonucleolysis. **a** Sagittal MRI T2-weighted image showing degenerative disc changes and a large extruded disc herniation at the level of L4–5, compressing the dural sac. **b** The corresponding axial MR T2-weighted image shows the disc herniation located at a left para-median site, obliterating the lateral canal recess with compression of the dural sac and traversing L5 nerve roots. **c**, **d** Lateral and anteroposterior fluoroscopic images showing the needle inside the disc center

In our patients, ozone chemodiscolysis was administered to both contained and uncontained herniations with no migrated or sequestrated fragments, and in the presence of mild to moderate degenerative disc changes (Figs. 4, 5, 6, 7, and 8). The integrity of the annulus was not a critical issue, as the oxygen-ozone mixture disperses within the disc material and spreads around the herniated disc material in the epidural space, exploiting its biochemical actions as a powerful oxidant and dehydrating agent, also at this level. In addition, the concomitant periganglionic ozone infiltration in close proximity to the herniated disc material enhances the process of dehydration and volume reduction, while the anti-inflammatory properties of ozone and steroid act locally on the inflamed ganglion root and yield more clinical benefits and a good end result [30]. Most (91%, *n* 10) of our patients with extruded hernias (Figs. 4, 8, and 9) reported satisfactory outcome with no statistically significant difference compared to other types of disc lesions. These results are similar to those obtained from other similar studies [10, 30, 31].

As in most percutaneous disc techniques, the procedure should not be applied in sequestrated or highly degenerated discs, “i.e., Black discs; Pfirrmann grading of V.” Sequestrated fragments were separated and could not gain benefit from an intradiscal injection. Black discs are highly dehydrated with severe height reduction and are usually associated with poor prognosis and results. Moreover; intradiscal ozone injection may result in further discal thinning and degradation. Thus, in such cases, we usually perform an intraforaminal periradicular injection of ozone, steroid, and a local anesthetic to counteract the inflammatory process at the disc-root conflict.

In our daily clinical practice, our usual standard treatment inclusion criteria are a little bit different compared to our study, as we sometimes include patients with lower prognosis, especially those who refuse or are unfit for the surgery. The usual treatment strategy is to advocate a single-treatment session as the first choice and postpone the second injection for patients with a partial unsatisfactory response after at least 2 months of observation. This strategy guarantees good therapeutic effect and reduces the incidence of complications as well as the cost of treatment. Xu et al. reported that there was no significant statistical difference in the outcome of those patients treated with either one or two treatment sessions of a 1-month interval. They recommended one-session treatment strategy with at least 1-month follow-up before a possible second injection [32]; this is in agreement with other treatment strategies [8].

The clinical follow-up of our patients is usually obtained without imaging control. There were many imaging studies that reported no strong correlation between patient response and morphological changes on imaging; the patients may show significant clinical response with little or no changes in imaging. This confirms that the root ganglion compression is not the single cause of symptoms but the associated inflammatory reaction plays an important role in the pathogenesis of discal lumbosciatica [33]. We request follow-up MRI only in patients with worsening or changing symptoms to rule out the possibility of a septic discitis or the development of recent discal lesions at different levels. Studies that included both clinical and radiological follow-ups have reported a reduction of herniation volume in 63–96% of the patients at 6-month control [25, 34–36].

The main limitations of our study were a relatively small series number and short follow-up period with lack of control group of patients. However, our study results are comparable to the results of larger series, longer-term follow-up, and controlled studies [8, 9]. The results are promising in view of minimally invasive, cost-effective, and safe procedure that can decrease the need for surgery.

## Conclusion

Oxygen-ozone nucleolysis is a simple, cost-effective, and safe minimally invasive technique for the treatment of pain and disability due to contained and uncontained disc herniations, with a short recovery period. It can be considered as an intermediate treatment option between failed conservative treatment and surgery.
